# Sweden’s public health response to COVID-19: a qualitative study building on a realist approach

**DOI:** 10.1186/s12913-025-13603-x

**Published:** 2025-10-22

**Authors:** Zahra Afshar Hosseinabadi, Anders Brantnell

**Affiliations:** 1 Department of Civil and Industrial Engineering, Industrial Engineering and Management, Uppsala University, Ångströmlaboratoriet, Lägerhyddsvägen 1, Uppsala, 752 37 Sweden; 2 Student Research Committee, School of Management and Medical Information Sciences, Isfahan University of Medical Sciences, Isfahan, Iran; 3 Department of Women’s and Children’s Health, Healthcare Sciences and e-Health, Uppsala University, MTC-Huset, Dag Hammarskjölds väg 14B, 1 tr, Uppsala, 752 37 Sweden

**Keywords:** Public health response, Realist approach, COVID-19, Qualitative study, Sweden, CIMO framework

## Abstract

**Background:**

Sweden’s healthcare system, known for its equity and efficiency, faced criticism in the early months of the COVID-19 pandemic due to its high mortality rate compared to other Nordic countries. This study aims to explore the Swedish public health system’s response to emerging respiratory infectious diseases, particularly COVID-19, using a realist approach. It analyzes contextual factors, interventions, mechanisms, and outcomes to provide a comprehensive understanding of the response, including debates around voluntary versus mandatory measures and the protection of vulnerable groups such as older adults.

**Objective:**

To explore how and why Sweden’s socio-political and healthcare context influenced the interventions employed and their acceptability at both individual and collective levels.

**Methods:**

A realist approach was employed, combining qualitative data from semi-structured interviews with 11 public health experts, including researchers and infectious disease specialists. Data were analyzed using both deductive and inductive approaches and interpreted through the Context-Intervention-Mechanism-Outcome (CIMO) framework.

**Results:**

Key contextual factors included high trust in authorities, political decentralization, and the structure of the healthcare system. Notable interventions involved targeted vaccination campaigns, recruitment of retired healthcare workers, and increased digitalization. Mechanisms driving outcomes included trust, acceptance, and stakeholder engagement, which facilitated adaptation and acceptability of interventions. Interviewees highlighted challenges related to the timing and adequacy of measures, particularly for older adults in long-term care.

**Conclusion:**

While Sweden’s public health response was effective in several areas, it faced challenges due to decentralization, voluntary non-pharmaceutical interventions, and workforce burnout. The realist approach highlights the importance of context-sensitive mechanisms such as trust and cooperation, and the need for stronger coordination and tailored strategies for vulnerable populations. These findings provide valuable insights for strengthening public health systems and pandemic preparedness globally.

**Supplementary Information:**

The online version contains supplementary material available at 10.1186/s12913-025-13603-x.

## Background

The healthcare system in Sweden is widely recognized for its excellence in promoting good health and providing equitable healthcare services to its citizens [1]. For example, in 2017, Sweden reported the highest life expectancy among all Nordic countries and the lowest age-standardized DALY (Disability-Adjusted Life Years) rates [2]. Furthermore, the Swedish health system is founded on fundamental principles that prioritize human dignity, cost-effectiveness, and a commitment to help to achive equal access to healthcare, irrespective of socioeconomic status or geographical location [3].

Despite these strengths, Sweden experienced a notably high number of COVID-19-related deaths during the initial months of the pandemic [4, 5]. In the first year, its death rate was four to ten times higher than that of other Nordic countries [4]. These figures highlighted potential shortcomings in Sweden’s healthcare system response to an unexpected respiratory pandemic [6]. Internationally, Sweden also drew attention for adopting less stringent countermeasures [7]. The country pursued a softer, largely voluntary approach to managing COVID-19, reflected in its relatively low score on the COVID-19 Stringency Index [8, 9]. This approach involved fewer mandatory lockdowns, the continued operation of primary schools, open businesses, and the absence of a mask mandate for much of the pandemic, instead relying on public trust and individual responsibility [10]. In contrast, neighboring countries such as Norway and Finland quickly enacted strict restrictions. Sweden avoided full lockdowns, opting instead for advisory measures, limits on large gatherings, and the temporary closure of high schools and universities—decisions shaped by both legal and cultural constraints on coercive action [10].

Many studies have provided valuable insights into Sweden’s response to the COVID-19 pandemic, highlighting issues such as the disproportionate impact on immigrant communities [11], challenges in managing a national crisis [12], stress experienced by healthcare workers [13], adaptability within hospital systems [14], the high level of trust enabling soft measures [15], and difficulties in protecting elderly and frail populations [4]. While these studies shed light on critical aspects, they do not offer a complete picture of the pandemic’s complexities in Sweden. In particular, none have employed a realist approach [16] to systematically examine the contextual factors, public health interventions, underlying mechanisms, and outcomes associated with the pandemic response. A realist approach provides a way to bridge this gap by not only identifying what works but also explaining why and how specific interventions produce particular outcomes in given circumstances [16]. Existing research has used various terms to describe this perspective on public health research, such as a realist approach [17], realist program theory [18], or realist synthesis [16]. In this paper, we use the term realist approach. This approach makes it possible to uncover the interactions between Sweden’s unique socio-political and healthcare contexts and the strategies adopted during the pandemic.

The main objective of this study is to explore how and why Sweden’s socio-political and healthcare context influenced the interventions employed and their acceptability at both individual and collective levels. To achieve this, we aimed to: (1) identify context-specific factors shaping intervention choices; (2) examine the mechanisms through which these interventions affected outcomes; and (3) reflect on how these mechanisms contributed to an adaptable health system response.

## Methods

### Study design and setting

This study employs a cross-sectional design to examine the responses of the Swedish public health system to COVID-19. A cross-sectional approach was chosen as it allows for data collection at a single point in time, making it particularly useful for assessing the functioning of the health system during the pandemic. The study is set within the Swedish public health system, which is organized into 21 regions and 290 municipalities. Each region is responsible for providing healthcare services, including hospital care, while municipalities manage social services such as elderly care and public health initiatives. The Swedish government, through the Public Health Agency of Sweden (Folkhälsomyndigheten), plays a central role in overseeing pandemic measures, including guidelines, testing, and vaccination efforts.

### Data collection

We used purposive sampling, incorporating both maximum variation and snowball sampling techniques to ensure the selection of a diverse range of public health experts. To account for contextual variations, we included professionals from various sectors, such as researchers, infectious disease specialists, and public health experts. Participants were required to have a minimum of three years of relevant experience, with researchers specifically needing experience within the public health system. To maintain a high level of expertise, individuals who lacked relevant experience or had no prior work experience in the public health system were excluded from the study. Data collection continued until data saturation was reached. Saturation was defined as the point at which no new data relevant to the study objectives emerged [19]. During the interviews, we observed recurring themes related to mechanisms and contextual factors. After 11 interviews, no new mechanisms or contextual factors were identified. In total, 11 respondents from various parts of the Swedish public health system were included.

Data were collected through semi-structured interviews conducted in person (*n* = 7) or via phone (*n* = 4) between October 2022 and February 2023. Respondents received written informed consent forms in advance. For phone interviews, consent forms were sent via email, signed digitally, and returned before the interview. Given the absence of visual cues in phone interviews, additional attention was paid to tone, pauses, and probing questions to assess participants’ reactions and engagement. The interviews lasted an average of 60 min, ranging from 45 to 90 min. All interviews were audio-recorded with participants’ consent and transcribed verbatim for analysis.

A generic interview guide was used, covering respondents’ perceptions of: (a) contextual factors influencing the outcomes of public management measures during respiratory outbreaks, (b) implemented interventions for managing respiratory outbreaks, (c) mechanisms underlying these interventions, and (d) the outcomes of these interventions.

### Data analysis

To better understand the relationships between various factors, we employed a realist approach, which allows for an in-depth exploration of how and why public health interventions lead to different outcomes across contexts. This methodology is particularly suited to examining the underlying mechanisms that determine the success or failure of interventions, highlighting that effectiveness is inherently context-dependent [20, 21]. Realist approch seeks to answer the questions of what works, for whom, under what circumstances, and why [22], making it especially valuable in complex health and social care settings [23]. Unlike approaches that focus solely on whether an intervention works, realist approach explains how interventions interact with contextual factors to produce outcomes. It also facilitates the development of program theories—evidence-based hypotheses about how and under what conditions interventions are most likely to succeed [23].

Following a realist approach, we analyzed the data using direct content analysis based on the CIMO framework, which consists of four components: (C)ontextual factors, (I)nterventions, (M)echanisms, and (O)utcomes [18, 21]. This framework guided the development of a program theory on health system preparedness for respiratory outbreaks. To achieve a comprehensive understanding, we combined both inductive and deductive reasoning [18]. Deductively, for the interventions component of the CIMO framework, we drew on the WHO’s six building blocks of a well-functioning health system: health workforce, health information system, service delivery, medicine and equipment, governance and leadership, and financing [20, 22]. Interventions reported in this study were organized within these categories, not as an exhaustive list, but as illustrative examples discussed by respondents and structured for clarity. The remaining three CIMO components: context, mechanisms, and outcomes, were analyzed inductively.

All interview transcripts were coded systematically. Both authors independently read and coded the data, compared their coding outcomes, and resolved discrepancies through discussion until consensus was reached. We employed an iterative process to refine codes into broader categories, aligning them with the CIMO framework. MAXQDA18 was used to manage and organize the coding process, thereby enhancing transparency and rigor.

To convey the relative prevalence of perspectives in our findings, we used the terms *majority* (raised by more than half of the respondents), *many* (raised by more than three but fewer than half), and *some* (raised by two to three respondents).

Regarding researcher reflexivity, ZAH is a public health researcher with expertise in respiratory diseases and a focus on global healthcare, while AB is an implementation researcher specializing in new technologies, with extensive knowledge of the Swedish healthcare system and qualitative methods. To enhance the credibility of our findings [24], we employed two key strategies: (1) member checking, by sharing findings with respondents for feedback and making adjustments as needed; and (2) reflexivity practices to minimize bias. Specifically, ZAH maintained a reflexivity journal to reflect on her own perspectives and potential biases during data collection and analysis, while AB actively considered how his knowledge of the Swedish public health system might influence interpretation.

## Results

The study included 11 respondents (7 males and 4 females) with diverse professional backgrounds, including researchers, public health experts, infectious disease specialists, and consultants. Two respondents were affiliated with national-level institutions (National Authority 1 and 2 in Table 1). At the regional level, two respondents worked at the same authority (Regional Authority 1 in Table 1). Most respondents were based at academic institutions, including four universities (Uppsala University, Malmö University, Karolinska Institutet, and Lund University) and a global health organization. To protect respondents, the organizations are anonymized in Table 1. Although many respondents were affiliated with universities, they also served on various COVID-19-related committees, giving them experience in both administrative and research roles. This dual perspective provided a more in-depth understanding of the issues. Their work experience ranged from 10 to 40 years, and their ages varied from 43 to 70 years, reflecting a highly experienced group with significant expertise in public health, research, and infectious diseases (see Table 1 for details).

**Table 1:** Respondent characteristics

Respondent	Sex	Age	Type of organization	Work experience(year)	Position
1	Male	52	University 1	20	Researcher
2	Male	55	Regional authority 1	15	Infectious diseases specialist
3	Female	62	Regional authority 1	38	Coordinator/business developer
4	Male	43	Global health organization	10	Researcher
5	Male	44	University 2	15	Public health expert
6	Female	59	National health organization 1	20	Public health expert
7	Female	55	University 1	25	Researcher
8	Male	62	University 3	35	Researcher
9	Male	45	University 3	15	Researcher
10	Female	70	University 4	40	Infectious diseases specialist
11	Male	66	National health organization 2	38	Consultant

**Fig. 1:**
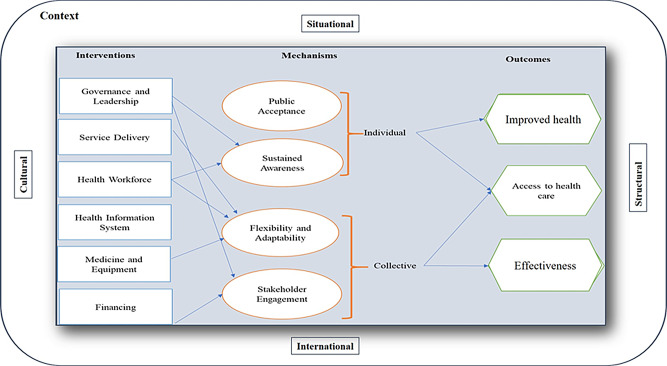
Program theory of Sweden’s public health system preparedness in the face of respiratory outbreaks

A realist approach examines the interactions between interventions and their context, the mechanisms that trigger them, and the resulting outcomes. This approach acknowledges that intervention effectiveness is not guaranteed, even when interventions are well implemented [18]. Figure 1 presents a program theory, developed based on our findings, on public health preparedness in response to respiratory outbreaks, building on the CIMO framework. The program theory illustrates the relationships between context, interventions, mechanisms, and outcomes, offering a comprehensive understanding of how various elements within the public health system interact. The figure highlights how contextual factors shape interventions, which are implemented across the six building blocks of the WHO public health system. These interventions, in turn, activate specific mechanisms that lead to outcomes such as improved health, better access to health services, and enhanced effectiveness.

In the following sections, we elaborate on the different components of the program theory and provide supporting quotes from respondents to illustrate our findings.

### Contextual factors influencing interventions

The contextual factors influencing the implementation of public health interventions were categorized into situational, structural, international, and cultural contexts.

#### Situational context

Public trust in healthcare authorities was an important factor, as the majority of respondents indicated that a high level of institutional trust in Sweden facilitated the acceptance of recommendations. As one respondent explained:

In Swedish society, trust in the government and community is generally high. Therefore, recommendations work quite well here… — Respondent 6

Another respondent added: “I believe that most of the time, people embrace the information and advice provided because they trust the authorities.” — Respondent 3.

Another important factor was demographic shifts, particularly the aging population in Sweden’s care facilities, which contributed to higher infection rates. Some respondents noted that Sweden had recently experienced a significant increase in its elderly population. This demographic shift, combined with the pandemic’s impact on long-term care facilities, led to higher infection and mortality rates, as one respondent highlighted:

Sweden’s growing elderly population, especially those in long-term care facilities, faced severe challenges during the pandemic. These care homes became centers of outbreaks, contributing to increased infection and mortality rates among residents. — Respondent 8

Many respondents also pointed out that healthcare decisions and lockdown measures varied by country, depending on factors such as size and population density. Each country had to tailor its approach to its specific circumstances, as one respondent stated:

I believe it’s crucial for each country to proceed based on its own facts. A certain country can’t treat this virus the same as others. For example, China, where it started, (is a big and densely populated country). However, Sweden found it very strange to completely shut down society. — Respondent 9

#### Structural context

Some respondents emphasized that political involvement and the decentralized structure of the healthcare system influenced pandemic outcomes. They highlighted that Sweden’s response was characterized by limited political engagement, reflecting the distinctive relationship between political leadership and public authorities. As one respondent noted:

The political dimension is often neglected. We had very little political involvement in the pandemic. It was not very common for the Prime Minister or the Minister of Health to address the nation. It was mainly the head of the Public Health Agency and the chief epidemiologist. — Respondent 5

Another respondent elaborated on this unique dynamic between politicians and authorities:

“In Sweden, there weren’t any politicians involved. There’s a unique relationship between Swedish politicians and Swedish authorities. I can’t think of any other country where politicians have as much faith and confidence in the authorities as they do here. — Respondent 6

While some respondents perceived decentralization as a challenge, particularly in terms of information sharing and coordination between system levels, others described it as beneficial, especially for local adaptability and responsiveness. In this regard, one respondent highlighted the challenges:

We have a multi-level healthcare system, and during the crisis, it was difficult for the information to flow through the system. The different levels found it challenging to communicate with each other. — Respondent 2

Another respondent explained the benefits:

I find the experiences with a decentralized model interesting, where health centers or hospitals have the initiative to adapt to change and handle the surge capacity. — Respondent 5

#### Cultural context

The majority of respondents stressed the importance of recognizing and accommodating cultural diversity during the pandemic response. They noted that a key barrier was the lack of accessible health information in multiple languages, which contributed to unequal outcomes. One respondent noted:

I’m not sure if all the local radio stations here were able to translate and carry the messages in other languages. As a result, the immigrant community was badly affected due to the lack of essential information. — Respondent 3

Additionally, many respondents highlighted a potential lack of awareness about disease transmission among immigrants with low socio-economic status, which made it challenging to communicate with these groups about the pandemic, as one respondent explained:

Some people from poor countries with limited education have little knowledge about viruses and bacteria. It’s challenging to explain these concepts to someone without basic training. — Respondent 1

#### International context

Some respondents noted that international cooperation, particularly through European vaccine procurement, contributed positively to Sweden’s approach to pandemic management, as expressed by one respondent:

I believe we achieved some of our vaccination goals through good collaboration with the European Union. This enabled us to receive vaccines on time and in sufficient quantities. — Respondent 8

In addition to joint vaccine procurement, some respondents also considered EU-level collaboration beneficial at a broader strategic level, as explained by another respondent:

We participated in EU-level discussions that helped coordinate strategies beyond just vaccine purchases. There were also shared approaches to data collection and vaccine monitoring. — Respondent 4

### Interventions

In addressing the second objective, we began with the six building blocks defined by the WHO, which are essential for ensuring a well-functioning healthcare system.

#### Health workforce

The majority of respondents stated that to address the shortage of healthcare workers during the pandemic, Sweden implemented interventions such as recruiting retired nurses and healthcare professionals, utilizing their expertise in vaccination efforts, and strategically deploying additional labor to meet the increased demand. This approach helped strengthen the healthcare system, as one respondent noted:

For vaccination, they recruited retired nurses and other healthcare professionals, which was a good way to identify and employ more nursing staff, addressing a critical aspect. — Respondent 5

The majority of respondents noted that to manage the increased demand during the pandemic, the hospitals reorganized their internal staffing and adjusted work schedules, as explained by one respondent:

Staff had to work extra hours, but they were compensated for it. They also rearranged staff within hospitals to address the increased demand. — Respondent 2

#### Health information system

Many respondents stated that to ensure easy access to accurate information during the pandemic, new communication channels were established, including digital tools, hotlines, and online platforms. These innovations aimed to reach people beyond traditional healthcare settings, as one respondent explained:

The welfare sector [i.e., the health services] quickly moved their services outdoors [i.e., outside the traditional healthcare settings] using digital tools for patient care. They also established communication channels where people could call in and receive accurate information. — Respondent 11

Another respondent added:

We [healthcare providers] used mobile apps, hotlines, and online portals. People could reach out anytime and get updates on symptoms or test centers. — Respondent 2

#### Service delivery

Many respondents explained that, to address service delivery challenges, interventions were implemented in Sweden’s public health system. These interventions aimed to enhance access, ensure continuity of care, prioritize safety, and improve quality, as stated by one respondent: “To overcome the disruptions caused by the pandemic, Sweden introduced targeted interventions in the public health system that focused on maintaining continuous care, and expanding access to essential health services.” — Respondent10.

The majority of respondents considered that access was improved through the introduction of a booking system for primary healthcare appointments, mass testing, and the availability of health advice through both primary healthcare centers and online sources, as explained by one respondent: “They made it a lot easier to get care by setting up a booking system for appointments, and making sure people could get health advice both at their local clinics and online.” — Respondent 6.

Many respondents stated that continuity of care was maintained via remote consultations, virtual support for patients, and the use of digital tools like telemedicine, and safety was prioritized by preventing sick individuals from meeting within nursing homes and transitioning services to outdoor settings, as explained by one respondent: “We kept things going by doing a lot of remote check-ins and virtual support, using telemedicine to stay connected.” — Respondent 11.

The majority of respondents stated that access to advice and health information was improved through primary care centers and digital tools, as one respondent explained:

People could call their primary healthcare center and receive advice on what to do. They also went online to gather more information. The system did well in terms of taking care of people and providing advice. — Respondent 3

#### Medicine and equipment

Some respondents stated that to enhance vaccination efficiency, Sweden focused on prioritizing vulnerable populations and monitoring the impact of the vaccination rollout on disease severity, hospitalizations, and mortality rates. Targeted vaccination campaigns were implemented to ensure a systematic and strategic approach to reaching those most in need. These efforts focused on efficiently using limited vaccine supplies and prioritizing vulnerable populations, as one respondent stated:

I appreciate the strategic and systematic approach taken through targeted vaccination campaigns. This ensures that the limited vaccine supply is utilized efficiently and reaches those who need it the most. It’s important to prioritize those who are most vulnerable and could benefit greatly from the protection offered by vaccinations. — Respondent 9

#### Governance and leadership

Many respondents stated that to address governance and leadership challenges during the pandemic, Sweden implemented several key interventions. These included fostering coordination across national and regional levels, promoting cooperation between communities and healthcare stakeholders, and maintaining open communication channels. Regular meetings were held to manage logistics and ensure the availability of medical supplies, as one respondent explained:

We held regular coordination meetings across national and regional levels, including logistics and procurement units, to ensure medical supplies were available where needed. These meetings helped anticipate shortages and redistribute supplies more effectively. — Respondent 7

Many respondents also emphasized that building networks and fostering cooperation between communities and regions strengthened regional collaboration, as noted by one respondent:

We established networks and cooperation between communities and regions during the pandemic. — Respondent 10

The majority of respondents stated that in terms of public health measures, Sweden implemented a partial lockdown while keeping schools open and encouraging remote work to limit transmission, as one respondent noted:

We had a partial lockdown, with schools still open. People were encouraged to work from home as much as possible. — Respondent 2

Some respondents stated that discussions around resource allocation and procurement played a key role in improving strategic planning and coordination during the pandemic, as explained by one respondent:

A lot of the planning worked better because ragions [regional] authorities really talked through how to share resources and get what was needed on time. That kind of teamwork made a big difference during the pandemic. — Respondent 2

Many respondents highlighted efforts to balance international collaboration with national preparedness, particularly in securing essential medical supplies. As one respondent explained:

We worked closely with the EU joint procurement scheme, but we also needed to build our own national reserves to manage local demands. — Respondent 6

Another respondent emphasized the importance of prioritizing critical resources early in the response:

In the early stages, our public health system faced uncertainty about supply chains. Strategic procurement discussions allowed us to prioritize essential items like PPE. — Respondent 10

#### Financing

Many respondents stated that to address financial challenges, Sweden introduced interventions such as providing financial and social support. Health insurance policies were adjusted to support individuals in staying at home, helping to reduce economic barriers that might otherwise have hindered adherence to public health guidelines. As highlighted by one respondent:

They made it easier for people to stay at home by changing the health insurance, providing 80% of income even during the first two days of sick leave. — Respondent 6

While this policy likely alleviated some financial pressure, some respondents respondents acknowledged that multiple economic and social factors—such as job insecurity and housing conditions—continued to influence people’s capacity to follow guidelines. As one of the respondents stated:

Even with support in place, people worried about losing their jobs or affording rent. That made it hard for them to fully follow guidelines. — Respondent 11

### Mechanisms activated by interventions

The third objective focuses on the mechanisms that led to the intended outcomes. In realist approach, mechanisms go beyond simple relationships; they aim to understand the deeper workings of how and why certain conditions lead to specific outcomes. To better analyze these mechanisms, we have classified them into two main categories: individual mechanisms and collective mechanisms. Individual mechanisms focus on the internal processes, behaviors, or decision-making of individuals, while collective mechanisms examine the interactions and dynamics between individuals or groups within a social context, such as cooperation or conflict, that shape outcomes at the collective level.

#### Individual mechanisms

We categorized public acceptance and sustained awareness as individual mechanisms because they primarily depend on the actions and attitudes of individuals within the population. By implementing interventions such as providing accurate information, fostering respectful communication, and addressing individual beliefs, public acceptance of measures and guidelines related to public health increased. Clear policy guidance and effective communication strategies helped build trust and promote compliance among the population. The majority of respondents emphasized that public trust in Swedish health authorities was key to achieving voluntary adherence to pandemic guidelines. Instead of relying on enforcement, authorities were able to influence behavior through credibility and public confidence in their intentions, as one respondent explained:

People didn’t need to be forced, they followed the advice because they believed the health agencies had their best interest in mind. — Respondent 3

However, this trust was not only institutional (trust in authorities and government) but also relational (trust in information sources and healthcare workers). As one respondent noted: “People really depended on their healthcare workers and trusted the info coming directly from them to feel confident and safe.” — Respondent 5.

Many respondents considered that efforts to improve healthcare service delivery through digital innovations, such as remote consultations and online tools, contributed to sustained awareness among the population during the pandemic. These solutions made healthcare more accessible and helped keep people consistently informed about how to manage their health. As one respondent explained:

Efforts to improve healthcare service delivery, such as remote consultations and digital tools, have significantly increased awareness among the population. — Respondent 3

#### Collective mechanisms

We categorized stakeholder engagement and adaptability as collective mechanisms because they involve coordination, collaboration, and collective decision-making among multiple actors. The majority of respondents emphasized that stakeholder engagement was fostered through close coordination with healthcare providers and other relevant actors. Engagement efforts included establishing networks between communities and regions, holding regular meetings across national, regional, and municipal levels, and involving a broad range of sectors. Transparent communication about operational challenges further enabled stakeholders to stay informed and adapt effectively. As one respondent remarked:

We actively engaged healthcare providers and relevant stakeholders through regular meetings and established networks between communities and regions. We had also meetings with different sectors on the national level and also on regional and municipal levels, and even the military were involved… — Respondent 8

Another respondent added:

Open communication about challenges, such as supply delivery interruptions, allowed stakeholders to be aware of the situation and adjust plans accordingly. — Respondent 7

The majority of respondents claimed that interventions such as task shifting, delegation of responsibilities, and the utilization of retired healthcare professionals enhanced the flexibility and capacity of the health workforce. These strategies allowed the healthcare system to respond more effectively to increased demand during the pandemic. As one respondent highlighted:

By delegating tasks and strategically reallocating responsibilities, we were able to respond to the increased demand during the pandemic. — Respondent 1

Another respondent stated:

Through task shifting and the utilization of retired healthcare professionals, we have successfully maximized the available resources. — Respondent 3

### Outcomes

The final objective focused on the outcomes of the interventions. These outcomes were categorized into three main areas, which are detailed below.

#### Access to healthcare

Many respondents explained that the interventions implemented fostered collaboration among government agencies, healthcare providers, community organizations, and other key stakeholders to improve social equity in healthcare. This broad engagement enabled stronger coordination and helped remove barriers, ensuring timely and equitable access to services such as testing, treatment, and vaccination, particularly for low-income and vulnerable populations.

I believe social equity plays a crucial role in improving public health, particularly for low-income groups… improved timely and equitable access to healthcare requires strong coordination among government agencies, healthcare providers, and community organizations. Through collaboration, we were able to remove barriers and make healthcare services more accessible, especially for vulnerable populations. — Respondent 7

#### Improved health

During the pandemic, various measures such as vaccination campaigns, physical distancing, and mask mandates were introduced to limit the spread of infection and protect vulnerable populations. These interventions were implemented not only to reduce transmission but also to prevent health systems from becoming overwhelmed. Respondents highlighted that the success of such measures depended heavily on public acceptance. When people understood the rationale behind them, such as the role of vaccination in building community immunity, they were more likely to comply voluntarily. This collective willingness to follow guidance was seen as a crucial factor in improving health outcomes. As one respondent explained:

When people understood the importance of vaccines and distancing they complied without needing enforcement and stayed at home to prevent spreading disease. — Respondent 5

Second, many respondents considered that sustained awareness was another crucial factor in shaping positive health outcomes. Ongoing communication and education campaigns by health authorities kept the public well-informed about the evolving situation and recommended preventive measures. This continuous awareness helped foster a collective sense of responsibility, supporting adherence to guidelines and promoting health equity by protecting vulnerable groups. As one respondent stated:

Preventive measures and raising awareness help minimize side effects and ensure better overall outcomes. This approach not only supports government efforts but also helps protect vulnerable groups, promoting equity in public health and safeguarding the entire population. — Respondent 8

#### Effectiveness

Respondents explained that the health system’s effectiveness during the pandemic depended on its capacity to adapt to rapidly changing conditions. Flexibility allowed health authorities to reallocate staff and resources where they were most needed, which helped maintain continuity of care and avoid critical gaps in service. Adaptability also meant that treatment pathways could be adjusted quickly to meet emerging demands, such as surges in COVID-19 cases. Coordination across levels of government ensured that policies were implemented consistently, while trust in public health authorities fostered cooperation among providers and the public. Together, these factors explain why the system was able to remain functional and responsive under pressure, which respondents viewed as the foundation of effectiveness in crisis conditions. As one respondent explained:

The adaptability of our healthcare system was crucial. We continuously adjusted our strategies based on new evidence and emerging challenges. This flexibility allowed us to reallocate resources efficiently, improved that patients received timely care and that our system remained responsive throughout the pandemic. — Respondent 3

To summarize the findings, the effective implementation of public health interventions in Sweden during the pandemic was influenced by an interplay of situational, structural, cultural, and international factors. High trust in authorities enable the acceptance of health guidelines, while demographic shifts, such as an aging population, contributed to increased vulnerability to infections. Decentralization in healthcare, political neutrality in pandemic management, and cultural diversity presented both strengths and challenges. Key interventions included recruiting retired healthcare professionals, enhancing digital communication, prioritizing vulnerable populations for vaccination, and fostering stakeholder collaboration. These efforts contributed to improved access to health services, increased public awareness, and overall system adaptability, leading to a more effective pandemic response.

## Discussion

This study contributes to understanding how Sweden’s unique socio-political context—characterized by high trust, decentralization, and soft governance—shaped public health interventions and their acceptability during the COVID-19 pandemic. For example, the dual nature of decentralization emerged as a key contextual factor influencing Sweden’s pandemic response. According to Bossert and Mitchell [25], decentralization in Sweden enabled flexibility and local adaptation, allowing regional and municipal authorities to adjust interventions to their specific circumstances. However, decentralization also complicated national coordination, creating challenges for unified information sharing and consistent policy enforcement [23]. These tensions echo findings from other decentralized health systems, where balancing local autonomy with national coherence remains a persistent challenge during public health crises [26]. Overall, our findings contribute to ongoing debates about how decentralization shapes pandemic governance and effectiveness.

In addition, public trust in healthcare authorities emerged as a pivotal element. Consistent with research from Norway and Belgium [23, 26–28], Sweden’s high level of public trust in government and healthcare systems contributed to the acceptance of guidelines and recommendations. This trust was crucial, as it led individuals to perceive public health measures as credible and aligned with their best interests.

Demographic factors, particularly the aging population in long-term care facilities also, played a significant role. The pandemic’s impact on the elderly underscored the importance of effective vaccination strategies. Population density also influenced disease transmission risk [29], necessitating stricter measures such as social distancing and restricted healthcare access in densely populated areas. In smaller countries with limited healthcare resources, careful allocation of interventions is essential to maximize impact [12]. For instance, while densely populated countries like China implemented strict lockdowns, Sweden adopted a more context-specific, less restrictive approach.

Our analysis of interventions through the lens of the WHO’s six building blocks reveals how Sweden’s strategies, such as workforce task shifting, financial compensation, and digital health innovations, activated mechanisms of adaptability and cooperation. These findings align with international trends showing that flexible workforce management and digital tools were pivotal in maintaining healthcare system functionality during COVID-19 [30, 31]. Importantly, Sweden’s reliance on voluntary compliance and recommendation-based governance contrasts with countries that enforced strict lockdowns, fueling ongoing debates about the trade-offs between individual freedoms and collective safety [32].

The interventions enabled the maintenance of access to healthcare services, the acceptance of public health measures and effective implementation of response measures. Digital tools and flexible strategies allowed Sweden’s healthcare system to adapt to challenges while maintaining service delivery. The country’s ability to adjust its strategies in real time contributed to efficient resource use and positive health outcomes. Flexibility and adaptability, linked to resilience [33, 34], were key features of Sweden’s governance system. A study conducted at a Swedish hospital during the pandemic further emphasized the importance of adaptive management practices, resource reallocation, and regional collaboration in balancing surge capacity and demand [14].

Public acceptance of health measures, particularly vaccination, was a critical individual mechanism. Evidence shows that compliance with guidelines helped reduce virus transmission and improve public health outcomes. During COVID-19, vaccine acceptance varied widely across countries, ranging from 17% to 67% [35]. Collective mechanisms, such as stakeholder engagement and adaptability, were also essential for coordination and smooth implementation. Lessons from the Ebola epidemic in Liberia highlight the importance of community engagement and local ownership in public health responses [36]. Similarly, in the United States, collaboration with local leaders strengthened community engagement during pandemics [37].

Despite these achievements, Sweden’s pandemic response drew substantial international and domestic criticism. Internationally, critics argued that its less restrictive approach may have contributed to higher mortality rates during the early phases of the pandemic, particularly in comparison to neighboring countries that adopted stricter lockdowns. Domestically, some public health experts and citizens questioned the adequacy of protective measures in eldercare facilities and debated the balance between individual freedom and collective safety [38]. Critics also emphasized challenges in national coordination caused by decentralization, which complicated communication and the consistent enforcement of policies across regions [39]. These criticisms highlight ongoing debates about the trade-offs inherent in Sweden’s public health strategy and underscore the importance of context-specific pandemic governance.

We believe that this study contributes to the development and application of a realist approach by demonstrating how contextual factors, mechanisms, and interventions interact within a health system. By mapping these dynamics, our findings highlight the contextual conditions and mechanisms most relevant to impactful public health responses. Moreover, the study illustrates how a realist approach can generate transferable knowledge, offering a structured framework for understanding the complexity of health system decision-making and for guiding future research and policy development in similar settings.

Additionally, these findings underscore the necessity to view health policy not in universal terms but through the prism of a country’s unique institutions, political traditions, and civic culture. Decentralization, in particular, played a dual role, providing flexibility at regional level but complicating national coordination, an issue similarly noted in recent analyses of COVID-19 governance [40, 41].

### Limitations

Although this study provides valuable insights into public health measures in Sweden during the COVID-19 pandemic and develops a program theory based on its findings, certain limitations should be considered when interpreting the results. First, the interviews included public health experts from various organizations but did not cover all 21 regions. As a result, regional differences in perspectives and experiences may not be fully represented, which could affect the generalizability of our findings across the entire Swedish health system. Second, selecting a substantial portion of relevant experts for interviews was challenging due to the complexity of the topic. While requiring a minimum of three years of experience ensured a high level of expertise, this criterion may have excluded individuals who played active operational roles during the pandemic, thereby narrowing the range of practical insights captured. Consequently, the findings may emphasize strategic and policy-level perspectives more than frontline implementation experiences.

Third, the use of the CIMO framework structured our analysis and facilitated the identification of key mechanisms, contextual factors, and interventions. However, the framework also inherently focused attention on aspects aligned with its components, which may have limited the identification of additional factors outside its scope. Similarly, while the study explored mechanisms and contextual factors shaping Sweden’s pandemic response, it did not directly address broader controversies, such as outcomes in long-term care facilities or the timing and scope of mandatory interventions. These gaps highlight areas where caution is warranted when applying the findings to policy evaluation or cross-country comparisons.

Despite these limitations, the study provides a systematic and theory-driven lens for understanding health system resilience and governance. Future research could build on these findings by surveying a larger, regionally representative sample or conducting in-depth interviews with a broader range of public health professionals involved in operational decision-making, thereby enhancing the applicability and depth of insights into complex public health responses.

## Conclusion

This study explored how Sweden’s socio-political and healthcare context shaped its response, using a realist approach and the CIMO framework. Drawing on qualitative insights from public health experts, the study shows how decentralization and public trust can both enable flexibility and adaptation while simultaneously creating challenges for coordination during crises. Trust in public health authorities proved essential for the acceptance of measures such as vaccination, underscoring the role of civic culture in shaping compliance. The findings demonstrate how contextual factors like decentralization and trust activate mechanisms, such as cooperation, adaptation, and adherence, that influence public health outcomes. These results highlight that health policies cannot be understood in universal terms but must be interpreted through the lens of each country’s unique institutions, political traditions, and civic culture.

## Supplementary Information

Below is the link to the electronic supplementary material.

Supplementary Material 1

## Data Availability

The datasets generated during and/or analysed during the current study are not publicly available to protect the anonymity of our respondents, but are available from the corresponding author on reasonable request.

## Data availability

The datasets generated during and/or analysed during the current study are not publicly available to protect the anonymity of our respondents, but are available from the corresponding author on reasonable request.
