# Supplementary material for: Sweden’s public health response to COVID-19: a qualitative study building on a realist approach
Source: BMC Health Serv Res. 2025 Oct 22;25:1393. doi: 10.1186/s12913-025-13603-x (PMC12542070; doi:10.1186/s12913-025-13603-x)
Supplement: Supplementary file 1 — Supplementary Material 1 [file 12913_2025_13603_MOESM1_ESM.docx]

**Appendix 1. The interview guide**

**Code: Interview Date: Interview Location:**

**Age**: Less than 30 30 to 40 41 to 50 Over 50

**Education:**

**Organization of Employment: Position:**

**Work Experience**: Less than 5 5 to 10 11 to 15 16 to 20 21 to 25 25 to 30

The main goal of this study is to develop a model for strengthening the public health system during the pandemic of new emerging respiratory diseases. (The term 'public health system' is related to disease prevention and community health promotion.) Since strengthening the public health system will lead to achieving the primary goals of the healthcare system, this study aims to identify the challenges faced by the public health system, the interventions employed, and proposals for strengthening the public health system during outbreaks of new emerging respiratory diseases.

1-Could you please explain the challenges that Sweden's public health system faced when dealing with new emerging respiratory disease outbreaks, including SARS, MERS, influenza, and COVID-19?

2-What interventions have been implemented within Sweden's public health system to effectively address new emerging respiratory disease outbreaks like SARS, MERS, influenza, and COVID-19?

3-What recommendations do you propose to strengthen Sweden's public health system when responding to new emerging respiratory disease outbreaks?

4-How do these interventions within public health systems target desired outcomes when dealing with new emerging respiratory disease outbreaks?

5-What are the outcomes of strengthening public health systems when faced with new emerging respiratory disease outbreaks?

6-What external and internal contextual factors play a pivotal role in determining the success or failure of public health systems when managing emerging respiratory disease outbreaks?

7-Are you familiar with the six building blocks introduced by the WHO, including service delivery, leadership and governance, financing, health information systems, health workforce, and essential medicine and equipment? What are your thoughts on strengthening these building blocks?

8-Could you please provide information about someone related to this topic whom I can interview?
